# Multivariate Analysis of BOLD Activation Patterns Recovers Graded Depth Representations in Human Visual and Parietal Cortex

**DOI:** 10.1523/ENEURO.0362-18.2019

**Published:** 2019-07-18

**Authors:** Margaret Henderson, Vy Vo, Chaipat Chunharas, Thomas Sprague, John Serences

**Affiliations:** 1 Neurosciences Graduate Program, University of California, San Diego, La Jolla, CA 92093-0634; 2 Department of Psychology, University of California, San Diego, La Jolla, CA 92093-0109; 3 Department of Medicine, King Chulalongkorn Memorial Hospital, Chulalongkorn University, Bangkok 10330, Thailand; 4 Department of Psychological and Brain Sciences, University of California, Santa Barbara, CA 93106-9660; 5 Kavli Foundation for the Brain and Mind, University of California, San Diego, La Jolla, CA 92093-0126

**Keywords:** depth, encoding model, fMRI, intraparietal sulcus, MVPA, vision

## Abstract

Navigating through natural environments requires localizing objects along three distinct spatial axes. Information about position along the horizontal and vertical axes is available from an object’s position on the retina, while position along the depth axis must be inferred based on second-order cues such as the disparity between the images cast on the two retinae. Past work has revealed that object position in two-dimensional (2D) retinotopic space is robustly represented in visual cortex and can be robustly predicted using a multivariate encoding model, in which an explicit axis is modeled for each spatial dimension. However, no study to date has used an encoding model to estimate a representation of stimulus position in depth. Here, we recorded BOLD fMRI while human subjects viewed a stereoscopic random-dot sphere at various positions along the depth (*z*) and the horizontal (*x*) axes, and the stimuli were presented across a wider range of disparities (out to ∼40 arcmin) compared to previous neuroimaging studies. In addition to performing decoding analyses for comparison to previous work, we built encoding models for depth position and for horizontal position, allowing us to directly compare encoding between these dimensions. Our results validate this method of recovering depth representations from retinotopic cortex. Furthermore, we find convergent evidence that depth is encoded most strongly in dorsal area V3A.

## Significance Statement

Estimating the position of objects in depth is essential for human behaviors such as reaching and navigating in a three-dimensional (3D) environment. Single neurons in visual cortex appear to support these abilities by encoding the depth position of stimuli, however, only a few studies have investigated how depth information is encoded by population-level representations in the human brain. Here, we collected fMRI data and used two multivariate analysis methods to examine the accuracy of depth encoding in retinotopic visual cortex. Our results show that depth representations are widespread in retinotopic cortex, with most accurate and robust encoding in intermediate dorsal region V3A. These findings are in agreement with past work, and may inform future studies of human 3D spatial perception.

## Introduction

The ability to perceive the location of objects in three-dimensional (3D) space is an essential component of the human visual system, and supports complex behaviors such as navigation through the environment and the guidance of eye and limb movements. However, although much is known about how 2D (i.e., retinotopic) space is encoded in the human brain, the encoding of depth representations is less well understood.

One of the canonical findings of early neuroimaging work in humans is the detailed description of large-scale, topographic maps of 2D space in occipital and parietal cortex (Fox et al., 1987; Sereno et al., 1995, 2001; DeYoe et al., 1996; Silver and Kastner, 2009). This detailed knowledge of spatial representations across cortex has enabled researchers to build encoding models that can accurately estimate 2D spatial locations from fMRI data obtained from a single visual field map (Naselaris et al., 2009; Sprague and Serences, 2013; Ekman et al., 2017; Vo et al., 2017). These encoding models estimate the spatial selectivity of single voxels to stimuli arrayed across 2D space, which amounts to finding a mapping from stimulus space to fMRI responses. A complete, well-estimated model combines information from voxels selective to all portions of space and is able to then invert the mapping accurately. That is, given a novel set of fMRI responses, this approach can generate a model-based representation of the spatial properties of the stimulus that the subject viewed on that set of trials (Serences and Saproo, 2012; Naselaris and Kay, 2015; Sprague et al., 2015).

A recent high-field fMRI study reported that binocular disparity selectivity is systematically organized in human cortex in a manner similar to orientation columns (Goncalves et al., 2015). This was significant in dorsal V2 and V3, but especially prominent in V3A and V3B/KO. The specialization of dorsomedial cortex for depth representations is consistent with previous human studies suggesting that perceptually relevant depth processing occurred in these same regions (Backus et al., 2001; Tsao et al., 2003; Bridge and Parker 2007; Preston et al., 2008). This systematic spatial organization may underlie the success of decoding studies that use multivariate pattern analysis (MVPA) to distinguish between stimulus positions in depth (Preston et al., 2008; Ip et al., 2014; Goncalves et al., 2015; Finlayson et al., 2017; Li et al., 2017). Taken together, these previous studies suggest that an encoding model for depth should also be able to accurately predict locations along the *z*-axis.

Here, we used both a support vector machine (SVM) classifier and a spatial encoding model to directly compare the representation of horizontal (*x*) and depth (*z*) information in different retinotopic regions of human visual cortex. Based on prior studies, we predicted that the SVM would be able to successfully classify depth information, especially in dorsomedial regions (Goncalves et al., 2015; Finlayson et al., 2017). Furthermore, we predicted that classifier discriminability would increase as the disparity between the two stimuli increased (Preston et al., 2008). We presented a wider range of disparities (out to ∼40 arcmin) than previous neuroimaging studies, which more fully samples the extent of human perceptual discriminability (Schumer and Julesz, 1984; Backus et al., 2001). We then used an inverted encoding model (IEM) to estimate the depth position of each presented stimulus. We predicted that earlier visual regions would be able to recover the depth position with some degree of precision, but that dorsomedial regions V3A, V3B, and IPS0 would show the highest quality depth representations.

## Materials and Methods

### Participants

Nine participants (seven female) were recruited from the University of California, San Diego community and completed the entire study. The number of participants was determined before data collection began based on the samples sizes used by fMRI studies with similar methodology (Barendregt et al., 2015; Goncalves et al., 2015; Vo et al., 2017). Participants were monetarily compensated for their participation and provided written informed consent in accordance with the human participants Institutional Review Board at University of California, San Diego. Each participant participated in two to four scanning sessions, each lasting 1–2 h. Data were also collected for three additional participants, but one dropped out of the study before data collection was complete, another was unable to fuse the binocular depth stimuli, and another discontinued the first session. These data were not analyzed, and are not reported here.

### Stimulus presentation equipment

**Figure 1.**
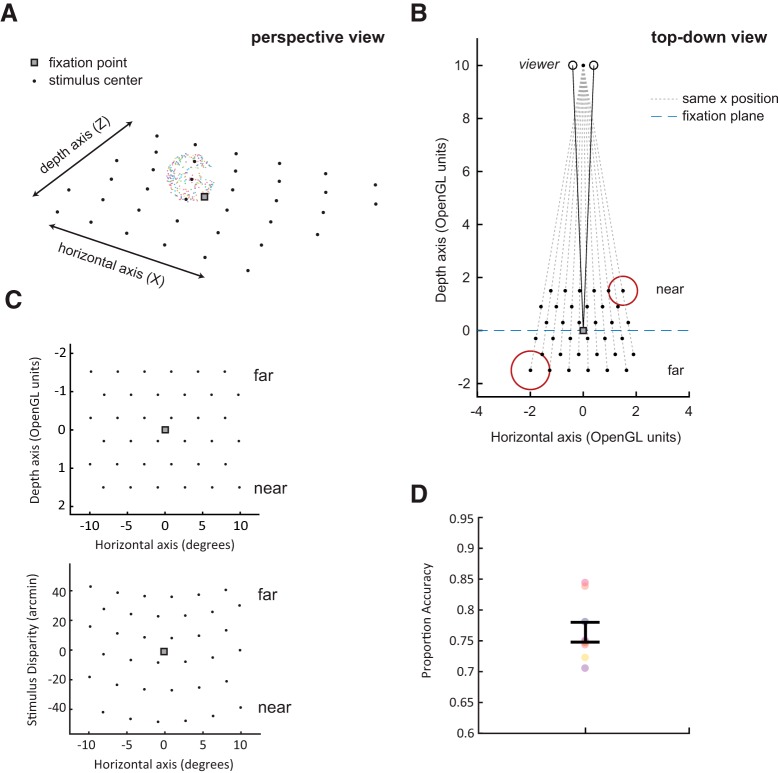
***A***, A perspective view on the grid of stimulus positions, and the stereoscopic sphere composed of colored dots, used to map position selectivity in retinotopic regions of visual cortex. The black points and the black box outlining the fixation point are for display purposes only, subjects only saw the sphere and the gray fixation point on a black background in the actual task. ***B***, The same example grid in OpenGL units. The size of the sphere, shown in red, was scaled with *z* position to maintain the same apparent size throughout. ***C***, Comparison of stimulus grid when plotted in units of physical position or disparity. Stimuli in each row of the grid share a physical *z* position (top panel), which results in a curved grid with nonlinear spacing when units are converted to disparity (bottom panel). This also results in a nonlinear spacing of the rows along the disparity axis. ***D***, Subjects performed a demanding contrast change detection task at fixation throughout all imaging runs; average accuracy on this task is plotted. Individual points indicate single subjects, error bars indicate mean ± SEM. For a plot of task performance broken down by the depth position of the stimulus, see Extended Data Figure 1-1.

Visual stimuli were presented to subjects using stereoscopic, MR-compatible video goggles (NordicNeuroLabs, VisualSystem) mounted on the head coil and lowered to fit comfortably in front of the subject’s eyes. Before the subject entered the scanner, the focus and pupillary distance of the goggles were adjusted so that the subject could comfortably fuse the images into a coherent stimulus. Displays were generated with the Psychophysics Toolbox extensions for MATLAB (Brainard, 1997; Pelli, 1997; Kleiner et al., 2007), using OpenGL software to render 3D stimuli. To generate a disparity between the two images, a different camera position was defined in OpenGL for each eye (left eye: [*x*, *y*, *z* = –0.4, 0, 10], right eye: [*x*, *y*, *z* = 0.4, 0, 10]), giving a screen-to-viewer distance of 10 units and an interpupillary distance of 0.8 units (Fig. 1). Frames were shown at a rate of 60 Hz, but since each frame was shown to each eye separately, this resulted in an effective frame rate of 30 Hz. Total screen size was set to 800 × 600 pixels, with an estimated vertical field of view (FOV) of 25° (throughout this text, ° refers to degrees visual angle, unless otherwise specified) and a horizontal FOV of 33° for average viewing parameters. We use these parameters to convert all the stimuli drawn in OpenGL units to an estimate of degrees of visual angle.

10.1523/ENEURO.0362-18.2019.f1-1 Extended Data Figure 1-1 Task performance was not significantly affected by disparity of the stimulus. Subjects performed a demanding contrast change-detection task at fixation throughout each imaging run, and performance did not change as a function of stimulus disparity. Black line shows mean ± SEM, colored lines show individual subjects. Download Figure 1-1, EPS file.

### Stimulus locations

During each run, the sphere stimulus appeared once at each of 36 different locations in the visual field (Fig. 1). The stimulus positions formed a staggered, triangular grid that evenly sampled locations along the horizontal (*x*) and depth (*z*) axes in a physical space rendered by OpenGL (Fig. 1). The depth positions ranged from 42.8 arcmin to –48.4 arcmin, and the horizontal positions ranged from 0.9° to 9.8° eccentricity in both directions. Spacing the stimuli evenly along both the *x*- and *z*-axes permitted us to estimate the encoding of both *x* position and *z* position using a spatial encoding model with the exact same basis set (see Inverted encoding models for spatial position). This allowed us to directly compare the spatial encoding quality across the *x*- and *z*-axes.

However, due to the nonlinear relationship between physical depth and disparity, this experimental design choice creates uneven spacing when these stimuli are replotted by binocular disparity (Fig. 1*C*). Additionally, because each row of the stimulus grid shares a physical distance, the rows become curved when plotted in units of disparity (Fig. 1*C*). This means that each row of the grid encompasses a small range of possible disparity values. Exact values of binocular disparity at each position are reported in Extended Data Table 1-1. Throughout this text, we group disparities according to *z* position, and refer to them according to the average disparity within each row (Table 1).

**Table 1.** Stimuli were presented at six unique *z* positions

*z* position (OpenGL)	Average disparity (arcmin)
–1.5	38.6
–0.9	25.6
–0.3	11.1
0.3	–5.2
0.9	–23.6
1.5	–44.6

Since disparity within each *z* position varied slightly with eccentricity (e.g., more peripheral positions appear further from the observer), we report the average value of disparity across each row of the grid shown in Figure 1. For individual values of disparity, see Extended Data Table 1-1.

Extended Data Table 1-1 Actual values of position in OpenGL space, degrees visual angle, and disparity for each position in the stimulus grid Download Table 1-1, DOC file.

Each stimulus was a sphere with an apparent 3.6° radius. To ensure that this apparent radius was constant across depth, stimulus sizes in OpenGL space were scaled such that the stimuli farthest from the viewer had an actual physical size larger than stimuli closest to the viewer (Fig. 1*B*). Sizes in OpenGL ranged from 0.73 units (furthest) to 0.55 units (nearest).

Because the grid was triangular, rather than rectangular, it was not perfectly symmetric about the *x*-axis. We chose to use a triangular grid because it more efficiently samples a plane than a rectangular grid. To maintain symmetry along the *x*-axis, grid locations were horizontally flipped on all even numbered runs such that 72 total grid locations (12 *x* locations × six *z* locations) were sampled between each pair of runs (Fig. 1). When generating inverted encoding model representations in the *x* dimension (see Inverted encoding models for spatial position), we reflected the data from the even numbered runs horizontally so that the *x* positions matched those of the odd-numbered runs, and averaged within each of the six remaining *x* positions.

### Task

During all functional scanning runs analyzed in this report, subjects performed an attentionally-demanding task at fixation. These “attend fixation” task runs were interspersed with runs of a separate task that required attention to the 3D sphere stimulus (“attend target” task). Data from the attend target task is available online. However, these data were much noisier, which we believe may be due to changes in vergence when attempting to simultaneously fixate and attend covertly in depth. Several participants reported difficulty maintaining consistent fusion during the covert attention task. Therefore, these data are not described here, nor do we plan to include it in future reports. For completeness, we give a description of both tasks below.

Both types of runs consisted of 36 trials, and lasted a total of 5 min (150 TRs). The fixation point was a 0.2° square continuously present throughout the run, surrounded by an 0.8° aperture in which no dots of the sphere stimulus were drawn. Each trial began with the appearance of the sphere stimulus for 3 s, followed by a jittered intertrial interval (ITI uniformly distributed between 2 and 6 s). Note that trial start times were not synced with TRs (TR = 2000 ms). To improve our ability to deconvolve the hemodynamic time courses, we added passive fixation at the beginning (2 s) and end (10 s) of the run. We also randomly interleaved 9 “null” trials per block in which no stimulus appeared for an entire trial and ITI. On attend fixation runs, the participant reported whether they detected a brief (200 ms) dimming or brightening of the fixation point with a key press (index finger for dimming, middle finger for brightening). On attend target runs, the participant reported the direction of a brief (500 ms) pulse of coherent motion in the sphere stimulus (clockwise or counterclockwise, when viewed from above). Both the fixation contrast event and the stimulus rotation event occurred on 100% of trials in both task conditions, always within the window of 1000–2500 ms after stimulus onset. No events occurred while the stimulus was not visible.

Participants performed between 7 and 21 repetitions of each task across all functional scanning sessions (mean ± SEM: 13.33 ± 1.76). This resulted in around 13 repetitions of each of the 36 stimulus positions, after aligning the positions between even and odd runs (see, Stimulus locations). Runs were always presented in pairs, with an attend target run followed by an attend fixation run. Mean accuracy on the fixation task was 76.4 ± 4.8%, and mean accuracy on the target task was 73.6 ± 12.9%. See Figure 1 and Extended Data Figure 1-1 for plots of performance on the fixation task.

### Sphere stimulus

The sphere stimulus consisted of a set of multicolored, flickering dots that were randomly positioned on the shell of a 3D sphere, with radius 3.4°. The color of each dot was defined by a vector in hue, saturation, value (HSV) color space, with each component in the range [0,1]. For all dots, we used fixed values for saturation (0.5) and for value (0.9). The hue of each dot was a randomly chosen position along the circular hue axis, resulting in a set of dots with different hues but the same saturation and value. These HSV vectors were passed into the MATLAB function hsv2rgb.m to generate an RGB triplet for each dot, which was used when rendering the dots through Psychtoolbox. Each dot had a lifetime of 100 ms and was redrawn every 100 ms at a random location on the sphere surface.

To generate a detectable motion signal, which participants responded to during attend target runs (data not reported, see Task), a proportion of the dots were rotated around the *y* (vertical)-axis to generate either clockwise or counterclockwise motion. When the stimulus first appeared, 100% of the dots were drawn in a random, flickering pattern with no coherent motion, then, during a 1-s interval during each trial, a proportion of the dots were selected to rotate. Rotating dots continued to rotate through the duration of their lifetime, but were re-drawn after 100 ms. Dots were rotated about the *y*-axis by 1.15° (in polar angle) per frame, with a frame rate of 30 Hz, resulting in a rotational speed of 34.38° (in polar angle) per second.

### Functional localizer task

To localize voxels that were responsive to the portion of the visual field in which stimuli were presented, a separate functional localizer task was used. This task consisted of 20 trials, and lasted a total of 5 min and 10 s (155 TRs). During each trial, a set of flickering, multicolored dots (rendered in same way as sphere stimulus, see Sphere stimulus) was drawn within a rectangular volume spanning one quadrant of the 3D visual field (left front, left back, right front, right back). Each dot field spanned the same 3D range of space in which spheres appeared during the main task; for example, the left front dot field spanned from –13.4° to 0° in *x*, from –3.6° to 3.6° in *y*, and from 0 arcmin to –44.6 arcmin in *z*. Each quadrant was stimulated five times per run, in a random order. Dots were onscreen for 8 s, and during this time a brief (200 ms) pulse of motion occurred in either the leftward or rightward direction. Subjects reported the direction of motion using either their index or middle finger.

Each subject performed between two and seven runs of the localizer task, which were interspersed during scanning sessions of the main task. This resulted in between 10 and 35 repetitions of each quadrant. Mean accuracy on this task was 79.2 ± 16.9%. All localizer runs for each subject were combined into a single generalized linear model (GLM) for analysis purposes.

### MRI acquisition

All participants were scanned using a 3T GE MR750 research-dedicated scanner at University of California, San Diego. We collected whole-brain functional images using a gradient EPI pulse sequence using a 32-channel head coil (Nova Medical) and an axial slice stack (19.2 × 19.2 cm FOV with 64 × 64 matrix, 35 3-mm-thick slices with 0-mm gap, TR = 2000 ms, TE = 30 ms, flip angle = 90°), which resulted in 3 × 3 × 3 mm^3^ voxels. For subject BJ, these data were collected with an eight-channel head coil (19.2 × 19.2 cm FOV with 96 × 96 matrix, 31 3-mm-thick slices with 0-mm gap, TR = 2250 ms, TE = 30 ms, flip angle = 90°) which resulted in 2 × 2 × 3 mm voxels. In the middle of the scan session, we acquired a B0 field map to correct for spatial distortions resulting from field inhomogeneities. During each scan session, we also collected a structural MRI using the same head coil as the functional data, accelerated by GE ASSET to obtain 1 × 1 × 1 mm^3^ voxels (25.6 × 25.6 cm FOV with 256 × 256 matrix, 172 1-mm-thick slices with 0-mm gap, TR = 8136 ms, TE = 3172 ms, flip angle = 8°).

### MRI preprocessing

The end result of our MRI preprocessing is an estimate of each voxel’s activation in response to each individual trial event. Voxels were subdivided into nine retinotopic regions of interest (ROIs). This process is described in detail below.

Each session’s structural scan was preprocessed to maximize the quality of alignment with the functional dataset. In BrainVoyager 2.6.1, we removed the head and skull tissue using automatic built-in algorithms. The images were then spatially resampled to a 1x1x1 mm^3^ resolution, and manually adjusted to align with the AC-PC plane. These data were then aligned to the subject’s higher-resolution structural image from their retinotopy session to allow for the use of retinotopically-defined ROIs (see below for more detail).

All functional data were unwarped using a custom script from the University of California, San Diego Center for Functional Magnetic Resonance Imaging written for FSL and AFNI. Each run was aligned to the same-session structural scan. We then performed slice-time correction, affine motion correction, and temporal high-pass filtering to remove slow signal drifts over the course of each session in BrainVoyager 2.6.1. These data were spatially transformed into Talairach space. Finally, the BOLD signal in each voxel was *z*-transformed within each run (after preprocessing, the resampled size of each voxel was 3 × 3 × 3 mm). Single-trial activation estimates, which were used for the SVM and IEM analyses described below, were obtained by taking an average of the *z*-scored BOLD signal at the 3rd and 4th TRs following stimulus presentation.

We followed previously published retinotopic mapping protocols to define the ROIs reported here (Swisher et al., 2007; Wandell et al., 2007; Jerde and Curtis, 2013; Winawer and Witthoft, 2015). Subjects viewed rotating wedge (10 cycles, 36 s/cycle), and bowtie stimuli (eight cycles, 40 s/cycle) presented at a single disparity through the MR-compatible video goggles (see Stimulus presentation equipment). To increase the quality of data from parietal regions, subjects performed a covert attention task on the rotating wedge stimulus (Sprague and Serences, 2013). They detected contrast dimming events in a row of the checkerboard (mean accuracy = 61.8 ± 13.9%). This stimulus was limited to 25.0° by 25.0° FOV.

After defining each retinotopic ROI, we applied a functional mask defined by voxels with significant BOLD response changes to any of the functional localizer stimuli (FDR corrected *q* < 0.05). The statistical parametric map for the localizer was defined by solving a traditional GLM convolving trial events with a canonical two-γ HRF (peak at 5 s, undershoot peak at 15 s, response undershoot ratio 6, response dispersion 1, undershoot dispersion 1). This limited our voxel population to those that were responsive to some position within the mapped *x-z* plane. In all analyses reported here, we used all voxels defined by the mask and did not equate voxel number across ROIs. When we repeated the major analyses with a fixed number of voxels in each region, we found similar results (data not shown).

The full range of retinotopic regions we examined was V1–4, V3A, V3B, IPS0-3, and LO1–2. After performing some initial analyses on this full list of regions, we found little evidence for depth representations in IPS1, IPS2, or IPS3. This may have been due to a limited number of voxels in each of these regions after thresholding with the localizer mask. Therefore, we do not include IPS1, IPS2, and IPS3 in the results reported here.

### SVM decoding

To assay information about stimulus position along the depth (*z*) axis, we used a linear SVM (libSVM software package, version 3.1, linear kernel) to classify trials according to their *z* position. We performed decoding between each individual pair of *z* positions (collapsing across *x* position within each row of the grid). Since our grid contained six distinct *z* positions, this gave 15 possible pairwise comparisons. For each of these comparisons, we identified all trials in which the stimulus was at one of the two positions of interest, and performed classification on this restricted data set.

To avoid overfitting, we used a leave-one-out cross-validation scheme, in which the model was trained on data from all but one imaging run, and tested on the left-out run. This process was repeated so that each run served as the test run once. We assessed decoding performance using d prime (d’), calculated using signal detection theory. We report d’ here in preference to classifier accuracy, because d’ is normally distributed and thus better suited for use in parametric statistics (Woolgar et al., 2015).

We measured the significance of decoding performance by generating a null distribution of d’. The null distribution values of d’ were generated by shuffling the position labels over all trials, and performing classification on this shuffled data set. This process was repeated over 1000 iterations within each subject and each ROI. We calculated significance for each ROI at the subject-averaged level, by calculating the mean d’ across subjects for the real data, as well as the mean d’ across subjects for each iteration of shuffling. A *p* value was then obtained by calculating the proportion of shuffling iterations on which the shuffled d’ exceeded the real d’, and the proportion on which the real d’ exceeded the shuffled d’, and taking the minimum value. We then performed FDR correction on the entire table of 150 *p* values (10 ROIs × 15 comparisons), at the 0.05 and 0.01 significance levels (Benjamini and Yekutieli, 2001).

To test whether pairwise *z* decoding performance differed significantly across disparity differences or across ROIs, we performed hierarchical model comparison in R (version 3.5.3) with the *lme4* package. We set up a nested series of six linear mixed regression models, specified as follows:

$$\begin{array}{l} M0:\;d’∼(1|subject) \end{array}$$

$$\begin{array}{l} M1:\;d’∼\Delta disp+(1|subject) \end{array}$$

$$\begin{array}{l} M2:\;d’∼\Delta disp+ROI+(1|subject) \end{array}$$

$$\begin{array}{l} M3:\;d’∼\Delta disp+ROI+(1|subject)+(1|ROI:\;subject) \end{array}$$

M4: d’ ∼Δdisp+ROI+(1|subject)+(1|ROI:subject)+(1|Δdisp:subject)

$$\begin{array}{l} M5:\;d’∼\Delta disp\text{ * }ROI+(1|subject)+(1|ROI:subject)+(1|\Delta disp:subject). \end{array}$$

Here, Δdisp is a continuous variable that we expect to have a linear relationship with d’. We used a likelihood ratio test to obtain a χ^2^ statistic comparing each model to the one below it in the hierarchy. The effects of disparity difference, ROI, and the interaction were assessed by comparing M1 and M0, M2 and M1, and M5 and M4, respectively. We performed pairwise comparisons between ROIs using the least squares means method, implemented through the *lsmeans* package in R. All *p* values were corrected using Tukey’s method.

Finally, to more closely examine the interaction between disparity difference and ROI, we estimated the slope and intercept of the d’/disparity relationship by performing resampling across subjects as described above. On each resampling iteration, we used linear regression to calculate a slope and intercept for the d’/disparity relationship within each ROI. To determine whether the slope in each ROI differed significantly from zero, we performed a two-tailed *t* test comparing the distribution of slopes against zero, and performed FDR correction across all ROIs (*q* = 0.01).

### Inverted encoding models for spatial position

In addition to using the linear SVM to decode depth information, we also trained a forward encoding model to get a continuous estimate of depth encoding. This model makes the assumption that the response of any individual voxel can be modeled as the sum of the responses of a set of underlying neural populations, or “channels,” each of which is sensitive to a particular set of locations in space (Brouwer and Heeger, 2009, 2011; Sprague and Serences, 2013). To model these channels, we created a set of basis functions that evenly tile the stimulus space (either the *x*- or the *z*-axis) and predicted how each basis function would respond to the stimulus on each trial. We then regressed the BOLD responses from each voxel onto these basis function responses, yielding a pattern of basis function weights for each voxel that describe its spatial sensitivity. Finally, using a separate set of test data, we used the estimated weights to predict the response in each spatial channel on each trial, and used this to generate a model-based spatial representation of the viewed stimulus.

First, we defined a general linear model to express the BOLD response of each voxel on each trial. This model is defined in Equation 1:

(1) $$B_{1}=WX_{1}$$

where $B_{1}$ is a matrix describing the mean activation of each voxel on each trial (*n* voxels by *t* trials), $X_{1}$ is a design matrix describing the activation in each spatial channel (*b* basis functions by *t* trials), and W is a weight matrix describing the transformation from channel space to voxel space (*n* voxels by *b* basis functions).

The design matrix $X_{1}$ was determined as follows. First, we defined a set of 6 basis functions that evenly tiled the dimension of interest (either the *x*- or *z*-axis). Basis functions were centered exponentiated cosine functions as described in Equation 2. This results in a curve with a Gaussian-like form, with a baseline of zero outside a fixed range.

(2) $$f\left(r\right)={\left(0.5\,\;\cos\left(\frac{r\pi }{s}\right)+0.5\right)}^{7}for\,\;r<s;0\,\;elsewhere$$

These were combined to yield matrix F (*b* basis functions by *p* pixels). To construct a design matrix $X_{1}$, we constructed a pixel representation of our stimulus on every trial (matrix S, *p* by *t* trials), and multiplied FS to yield $X_{1}$ (*b* by *t*). We can then use B_1_ and X_1_ to solve for the basis function weights using the Moore–Penrose pseudoinverse:

(3) $$\hat{W}=B_{1}{\left(X_{1}{}^{T}X_{1}\right)}^{-1}X_{1}{}^{T}$$

Finally, we inverted the forward model to decode stimulus position. For a matrix of voxel responses recorded during independent scanning runs, $B_{2}$ (*n* voxels by *t* trials), we can calculate the response of each channel on each trial, $\hat{X_{2}}$, by multiplying by the pseudoinverse of the estimated weight matrix:

(4) $$\hat{X_{2}}={\left({\hat{W}}^{T}\hat{W}\right)}^{-1}{\hat{W}}^{T}B_{2}$$

For visualization and fitting purposes, we multiply ${\hat{X_{2}}}^{T}$ by F to yield a *t* trial by *p* pixel matrix of model-based representations for each test trial. Training and testing were performed using a leave-one-run-out cross-validation scheme, so that each scanning run served as the test set exactly once.

For each subject, model-based representations of stimuli at the same spatial positions were averaged over all trials to obtain a mean representation of each stimulus position. In the *x* dimension, for all odd-numbered runs, we flipped the *x* coordinates of each stimulus position from left to right. This allowed us to average over stimulus positions that were equally far from fixation (see, Stimulus locations). In total, this resulted in six representations of *x* position and six representations of *z* position for each subject and ROI.

Each model-based representation was then fit with an exponentiated cosine function having parameters of center, size, amplitude and baseline (see Fits of IEM-based representations).

### Fits of IEM-based representations

Model-based representations of each stimulus position along the *x*- and *z*-axes were fitted with curves having the same form as the basis functions (Eq. 2), with additional parameters for baseline and amplitude:

(5) $$f\left(x\right)=b+a{\left(0.5\,\;\cos\left(\frac{\left|x-c\right|\pi }{s}\right)+0.5\right)}^{7}for|x-c|<s;0\,\;elsewhere$$

Fitting was performed using a two-step iterative procedure. First, to obtain an initial estimate of the center *c,* size *s,* amplitude *a*, and baseline *b,* we generated a grid over a subset of possible centers and sizes. Centers ranged from –2.75 to 2.75 units in OpenGL space (corresponding to the region of visual stimulation during the task) in steps of 0.1, and sizes ranged from 1.5 to 15 in steps of 0.1. Using each combination of center and size, we calculated the fit amplitude and baseline using linear regression, restricting amplitudes to lie between 0 and the maximum height of the representation (max value – min value). The fit with the lowest root-mean-squared-error (RMSE) was used as the starting point for an additional fine-tuning procedure, using a linear optimization algorithm to minimize the RMSE (*fmincon* from MATLAB’s optimization toolbox). During this fine-tuning step, center and size of the fit was each constrained to fall within one grid step (0.1 units) of its initial value, baseline was constrained between –5 and 5, and amplitude was again constrained between 0 and the maximum height of the representation (max value – min value). If this final step resulted in an increase in RMSE relative to the initial fit, then the initial fit was used, otherwise the parameters of the fine-tuned fit were used.

Although we estimated values for four fit parameters, we focus here on the estimates of center and amplitude. Both the center and the amplitude of model-based representation fits can be used as informative measures of representation quality. Baseline and size estimates are shown in Extended Data Figures 6-1, 6-2.

The accuracy of our model-based representations was evaluated in several ways. First, we assessed the correspondence between representation fit centers and actual stimulus centers. To generate estimates of the mean and 95% confidence intervals (CIs) for fit center across our sample, we used bootstrapping (Efron and Tibshirani, 1993). That is, we resampled with replacement across subjects 1000 times. This generated an empirical estimate of the variability of the data. Taking the absolute value of the difference between the fit center and the actual stimulus center gave us an estimate of the overall representation error, depicted in Figure 6*A* and Extended Data Figure 6-2*A*. We used the same method to generate bootstrapped means and 95% CIs for all other fit parameters (size, amplitude, and baseline), which are shown in Figure 6*B*, Extended Data Figures 6-1, 6-2.

After visualizing the distributions of model-based representation error, we formally tested whether this measure differed significantly across stimulus positions or across ROIs. Using the estimates of representation error for each subject (not bootstrapped), we constructed a nested series of linear mixed regression models and performed a likelihood ratio test using the *lme4* package in R. We set up a nested series of six linear mixed regression models, specified as follows:

$$\begin{array}{l} M0:\;\text{error}∼(1|subject) \end{array}$$

$$\begin{array}{l} M1:\;\text{error}\;∼position+(1|subject) \end{array}$$

$$\begin{array}{l} M2:\;\text{error}∼position+\text{(}1|position:subject)+(1|subject) \end{array}$$

$$\begin{array}{l} M3:\;\text{error}∼position+ROI+(1|subject)+(1|pos\text{i}tion:subject) \end{array}$$

$$\begin{array}{l} M4:\;\text{error}∼position+ROI+(1|subject)+(1|position:subject)+(1|ROI:subject) \end{array}$$

$$\begin{array}{l} M5:\;\text{error}∼position\text{ * }ROI+(1|subject)+\text{ (}1|position:subject)+(1|R\text{O}I:subject). \end{array}$$

Here, position is a categorical variable with six different levels. We compared each model to the one below it in the hierarchy using a likelihood ratio test. The effects of position, ROI, and the interaction were assessed by comparing M1 and M0, M3 and M2, and M5 and M4, respectively. We performed pairwise comparisons between ROIs using the least squares means method, implemented through the *lsmeans* package in R. All *p* values were corrected using Tukey’s method. We repeated the same procedure to perform an analysis on the fit amplitudes.

Finally, as a second measure of representation accuracy, we estimated a slope and intercept for the relationship between representation center and actual stimulus center. The logic behind this measure is that if the model-based representations from the IEM perfectly captured the stimulus positions, the slope would be close to 1. To estimate the variability of the slopes, we took the bootstrapped estimates of representation fit centers described above, and fit a line to every single resampling iteration. We then used the distribution of resampled slopes to obtain a *p* value comparing the slope in each ROI against zero. The resulting *p* values were FDR corrected across all ROIs for both *x* and *z* (*q* = 0.01). Additionally, we used the resampled slope distributions to perform pairwise comparisons between individual ROIs, followed by FDR correction (*q* = 0.01). The estimated slopes are plotted in Figure 6*C*.

### Code accessibility

All code and data required to reproduce the analyses of this paper is available on the Open Science Framework (https://osf.io/j7tpf/). Code files can also be downloaded directly (Extended Data 1). We performed all analyses on a Linux operating system.

10.1523/ENEURO.0362-18.2019.ed1 Extended Data 1 Analysis code can be accessed by downloading the zip file associated with this manuscript. See the README file in this folder for details. Download Extended Data 1, ZIP file.

## Results

### SVM decoding

**Figure 2.**
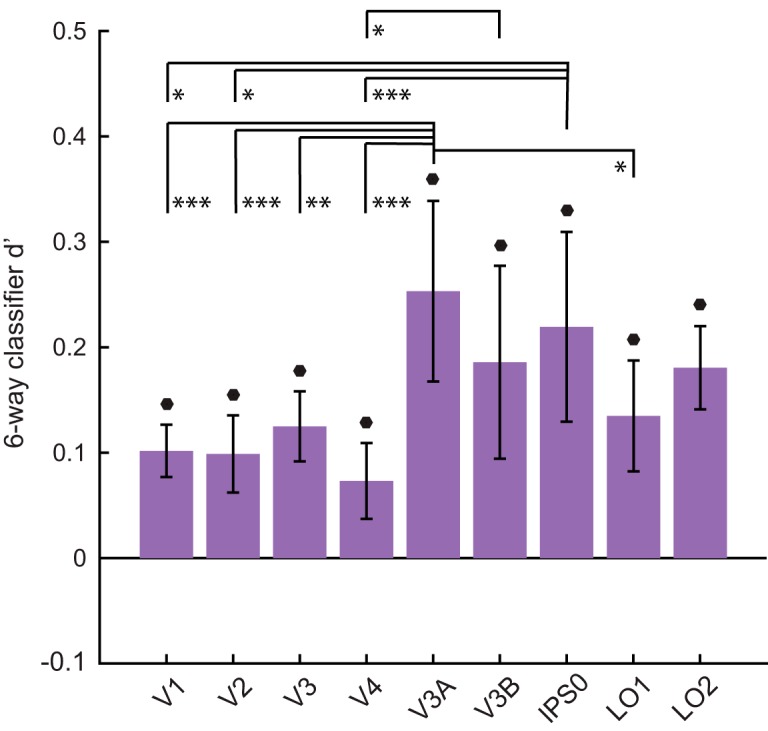
Average six-way decoding performance in the *z* dimension. Performance (d’) values were averaged across subjects and error bars indicate SEM. Filled circles over individual bars indicate above chance decoding after FDR correction at *q* = 0.01. Asterisks indicate significant differences at the 0.05 (*), 0.01 (**), and 0.001 (***) significance levels, respectively. Pairwise comparisons were corrected using Tukey’s method; for details, see Materials and Methods.

We first used a linear SVM as a benchmark to compare our results to previous work. We performed classification between each possible pairing of *z* positions (for six locations in depth, this resulted in 15 pairwise *z* decoding schemes). Averaging across these 15 comparisons gave a single value for six-way decoding, which was numerically highest in V3A, V3B, and IPS0 (Fig. 2), as has been found in past work (Preston et al., 2008; Goncalves et al., 2015). Although average decoding performance differed among ROIs, d’ was above chance in all regions examined, including early visual cortex.

**Figure 3.**
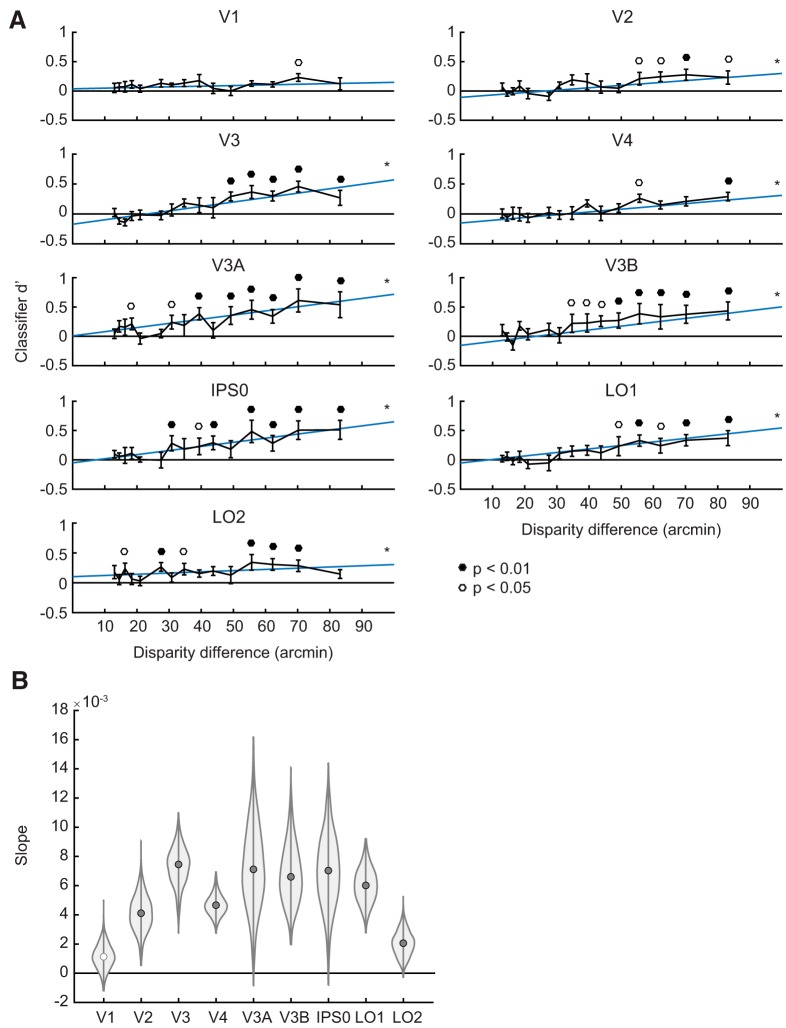
SVM classifier performance (d’) depends on difference in stimulus disparity between the positions of interest. ***A***, Each unique pair of the six stimuli in depth is plotted on the abscissa, with small disparity differences indicating that the stimuli were close together in the *z* dimension. Blue lines depict the fit to the mean bootstrapped across subjects, and asterisks (*) indicate a significantly positive slope (FDR *q* = 0.01). Filled and open circles above individual error bars indicate significance after FDR correction at *q* = 0.01 and *q* = 0.05, respectively. For the same data plotted as a dissimilarity matrix, see Extended Data Figure 3-1. ***B***, Bootstrapped distribution of slopes for the relationship between classifier d’ and the disparity difference between the positions of interest. Filled gray circles indicate slopes significantly above 0 (FDR *q* = 0.01).

Next, we examined performance on each individual pairwise discrimination, and plotted classifier performance as a function of the difference in disparity between the two positions of interest. Overall, this analysis revealed a positive relationship between the disparity difference between stimuli and associated decoding performance (Fig. 3; Extended Data Fig. 3-1). At the largest disparity difference (83.2 arcmin), seven out of nine ROIs showed above-chance decoding performance, with V3A showing the highest performance (accuracy = 60.0 ± 4.0%, d’ = 0.54 ± 0.22). Performance at the second largest difference (70.2 arcmin) was comparably strong, with eight out of nine ROIs showing above-chance decoding performance, and the highest performance again in V3A (accuracy = 61.3 ± 3.3%, d’ = 0.61 ± 0.20). As the difference in disparity between the two positions decreased, the number of ROIs with above-chance decoding decreased, with no ROIs showing above-chance discrimination of disparities <16.2 arcmin. Additionally, plotting pairwise decoding performance in matrix form (Extended Data Fig. 3-1) revealed that the nearest *z* position (–44.6 arcmin) tended to be the most discriminable from other positions.

10.1523/ENEURO.0362-18.2019.f3-1 Extended Data Figure 3-1 Dissimilarity between all pairs of Z positions. Color of each square indicates the performance (d’) of a linear classifier trained to discriminate between the positions of interest. Solid and open circles indicate above-chance decoding performance at the 0.01 and 0.05 significance levels, respectively. High values in the bottom row of each plot indicate that the nearest Z position (–44.6 arcmin) was the most easily discriminated from other positions. Download Figure 3-1, EPS file.

To evaluate whether *z* position decoding performance differed significantly across disparity differences or across ROIs, we performed hierarchical model comparison between several linear mixed regression models (for details, see Materials and Methods). This revealed a significant effect of disparity difference (χ^2^(1) = 155.76, *p* < 10^−15^), a significant effect of ROI (χ^2^(8) = 47.44, *p* < 10^−6^), and a significant interaction between disparity difference and ROI (χ^2^(8) = 37.42, *p* < 10^−5^). We further investigated the main effect of ROI by performing pairwise comparisons, which revealed several important differences. Decoding was significantly higher in dorsal area V3A than in early visual areas V1–V4 (Tukey corrected *p* < 0.05: V3A-V1 *t* = 0.15, SE = 0.03, *p* = 0.0005; V3A-V2 *t* = 0.15, SE = 0.03, *p* = 0.0003; V3A-V3 *t* = 0.13, SE = 0.03, *p* = 0.0071; V3A-V4 *t* = 0.18, SE = 0.03, *p* < 10^−4^), and was also higher in IPS0 than in V1, V2, and V4 (Tukey corrected *p* < 0.05: IPS0-V1 *t* = 0.12, SE = 0.03, *p* = 0.0208; IPS0-V2 *t* = 0.12, SE = 0.03, *p* = 0.0157; IPS0-V4 *t* = 0.15, SE = 0.03, *p* = 0.0009). Performance in V3B was also significantly higher than performance in V4 (*t* = 0.11, SE = 0.03, *p* = 0.0332).

Although decoding performance generally scaled positively with disparity difference, the strength of this relationship differed among ROIs. To investigate this interaction, we used bootstrapping to estimate the slope of the d’/disparity relationship in each ROI. Estimated slope was highest in V3, V3A, V3B, and IPS0, and was significantly higher than zero in all ROIs except for V1 (Fig. 3*B*).

### Inverted encoding model

In the next analysis, we used a forward encoding model to explicitly model a single continuous *z*-axis and tested how well we could estimate stimulus position from this model. As a comparison condition, we also modeled a single continuous *x*-axis. We built two separate encoding models: one for *x* position and one for *z* position. Each voxel was modeled as a linear combination of 6 modeled spatial channels with sensitivity along either the *x* or *z* dimension (see Materials and Methods). Importantly, the structure of the spatial encoding model was identical between the two dimensions, and the stimuli were evenly gridded along both the *x*- and *z*-axes (Fig. 1). These experimental design choices allowed us to directly compare the quality of *x* encoding and *z* encoding for positions in rendered physical space (i.e., OpenGL coordinates). After estimating the channel weights on a set of training data, we tested the accuracy of the model by inverting the weights and applying them to a novel set of test data. This yields an estimate of the stimulus position along the *x*- or *z*-axis, which we refer to as a model-based stimulus representation.

**Figure 4.**
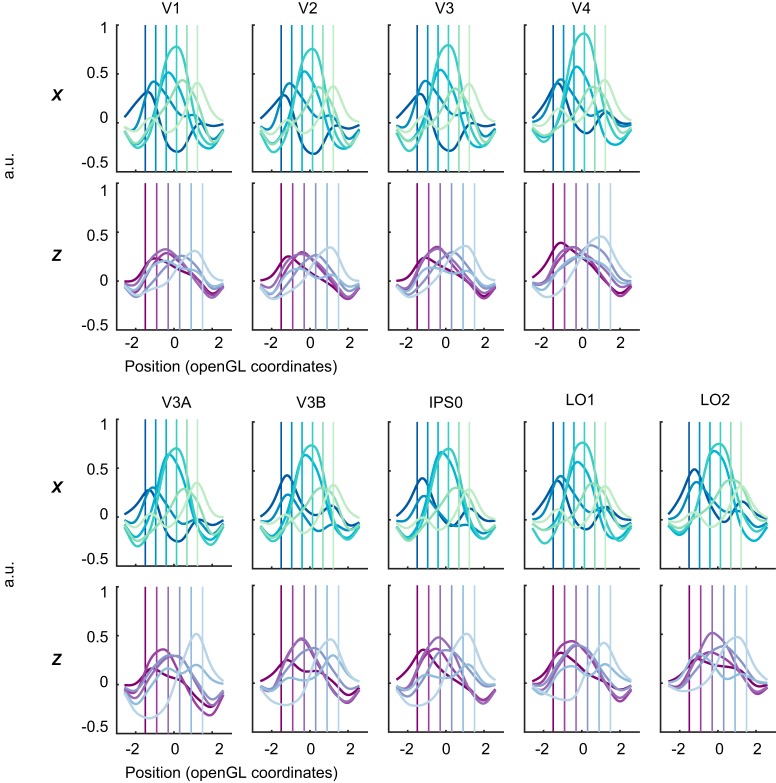
Average model-based representation of stimuli at each position along the *x* (blue-green) and *z* (purple) dimensions. Vertical lines indicate where the stimuli were presented along the *x*- or *z*-axis. The curved lines in matching colors indicate representations of the corresponding positions.

We plot the mean model-based representations for six positions along the *x*-axis and six positions along the *z*-axis in Figure 4. The *x* representations were our positive control, and were expected to show high accuracy based on previous reports (Sprague and Serences, 2013; Vo et al., 2017) and knowledge about retinotopic organization in these areas. We find that *x* representations are much more accurate than *z* representations, especially in early visual areas V1–V4. However, in dorsomedial areas the estimated depth positions appear to increase in accuracy. This can be seen by comparing the peaks of the curved lines in Figure 4 with the locations of the vertical lines in matching colors.

To quantify the model-based representations, each subject’s average representation was fit with an exponentiated cosine function to describe its center, size, amplitude, and baseline (Equation 5). We estimated the variability of each fit parameter by resampling with replacement across subjects (see Materials and Methods) to generate an empirical estimate of the 95% CIs. These distributions for all parameters are plotted in Figure 6, Extended Data Figures 6-1, 6-2. We focus here on the fit centers and amplitudes.

10.1523/ENEURO.0362-18.2019.f6-1 Extended Data Figure 6-1 Additional parameters of best-fit curves for model-based representations of stimulus X and Z position. ***A***, Fit baseline. ***B***, Fit size. In all plots, mean and confidence intervals across participants shown in black solid lines, with 95% CIs computed by bootstrapping. Individual participants are shown in colored circles. Download Figure 6-1, EPS file.

10.1523/ENEURO.0362-18.2019.f6-2 Extended Data Figure 6-2 Parameters of best-fit curves for model-based representations, plotted as a function of stimulus position along *z*-axis. Values are averaged across all brain regions. ***A***, Absolute value of fit center error. ***B***, Fit size. ***C***, Fit amplitude. ***D***, Fit baseline. In all plots, mean and confidence intervals across participants shown in black solid lines, with 95% CIs computed by bootstrapping. Individual participants are shown in colored circles. Asterisks indicate differences significance at the 0.05 (*), 0.01 (**), or 0.001 (***) significance level. Note that no pairwise tests were performed for panels ***B***, ***D***; for details, see Materials and Methods. Download Figure 6-2, EPS file.

**Figure 5.**
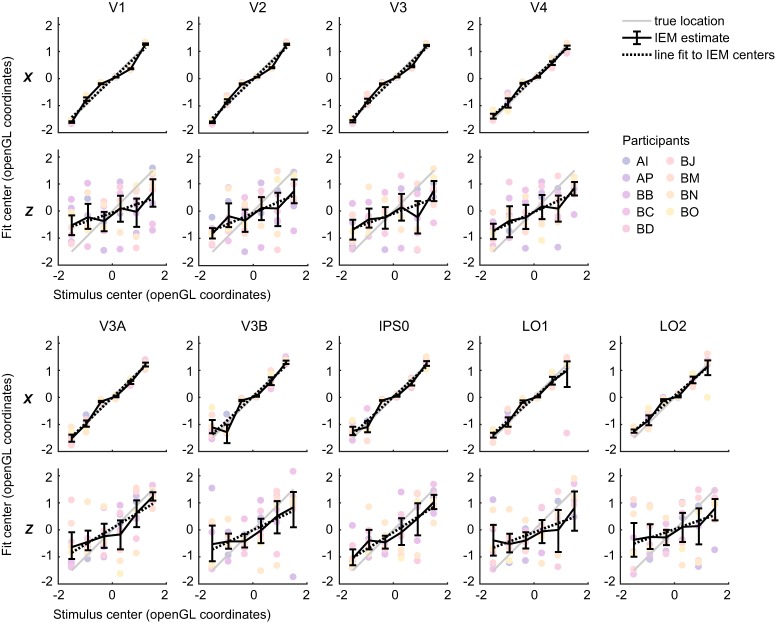
Best fit centers of model-based representations at each stimulus position versus actual displayed stimulus center (thin gray line). Average fit center across participants shown in black solid lines, with 95% CIs computed by bootstrapping. Individual participants are shown in colored circles. Mean linear regression solution is shown with a dotted black line, where high accuracy representations have a dotted line that overlaps with the gray line.

**Figure 6.**
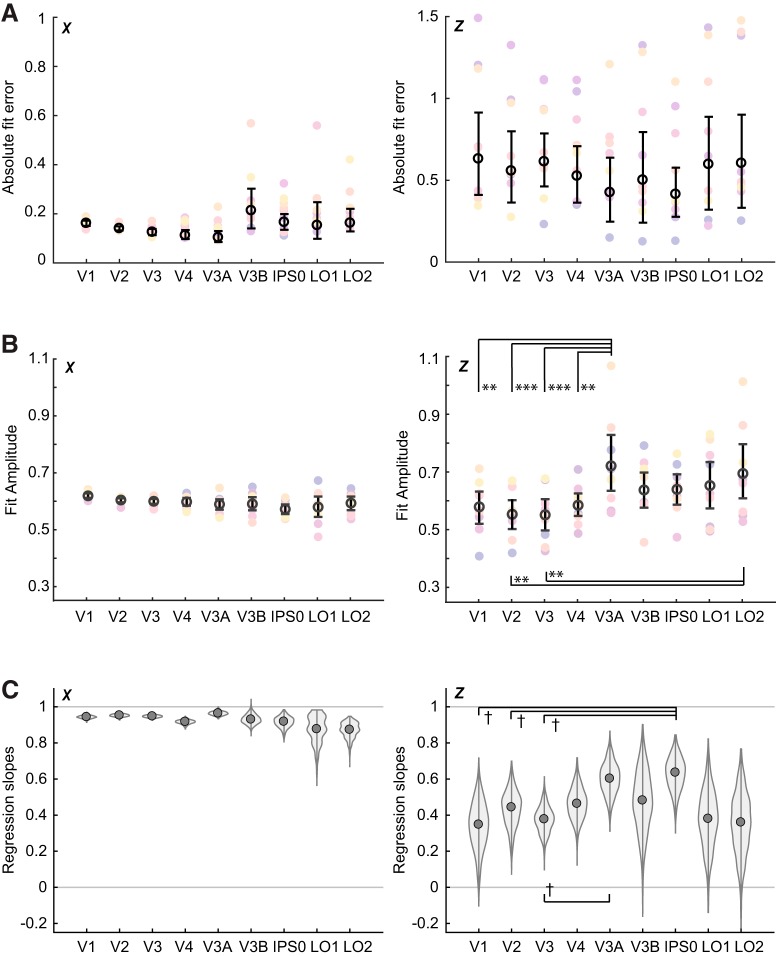
Quality of model-based representations assessed three ways. ***A***, Absolute error between the true stimulus position and the estimated position for both *x* (left) and *z* (right). ***B***, Best-fit amplitude of representations, averaged across position. In both ***A***, ***B***, open black circles and solid black lines indicate mean and 95% CIs computed by bootstrapping. Individual participants are shown in colored circles. Asterisks in the rightmost panels indicate differences significant at the 0.01 (**) or 0.001 (***) significance level. Note that no pairwise tests were performed for the left panels of ***A***, ***B***, ***C***. Bootstrapped distribution of linear regression slopes from data in Figure 5. This represents the intersubject variability of the slopes. Daggers (†) indicate differences significant at the 0.05 level before FDR correction. For plots of representation fit size and baseline, see Extended Data Figures 6-1, 6-2.

We first plotted the distribution of fit centers against the actual stimulus positions, allowing us to visualize the overall accuracy of the representations (Fig. 5). We also used the distribution of fit centers to estimate the absolute error of representations in each ROI averaged over positions, shown in Figure 6*A*. From these plots, it can be seen that in the *x* dimension, the representation centers generally track the stimulus location at all positions and ROIs. However, a few *x* representations deviated slightly from the true stimulus center in a statistically significant fashion. For example, the two leftmost positions were slightly misestimated by area V3B (signed error between fit and true positions 0.39 [0.18, 0.66]; –0.33 [–0.73, –0.05]), suggesting that some level of noise should be expected in the *z* representations as well. By comparison with the *x* data, the *z* representations were both less accurate (larger absolute error) and less consistent (larger CIs). IEMs based on data from dorsomedial regions V3A and IPS0 represented stimulus depth with the lowest error (Figs. 5, 6*A*). To test whether the position representation error was significantly different between stimulus positions or between ROIs, we used hierarchical model comparison of a nested set of linear mixed regression models (see Materials and Methods). This analysis revealed a significant main effect of position (χ^2^(5) = 36.55, *p* < 10^−6^), but no effect of ROI (χ^2^(8) = 10.75, *p* = 0.22), and no interaction between the two (χ^2^(40) = 35.09, *p* = 0.69). We further investigated the effect of *z* position by performing pairwise comparisons between all *z* positions, which showed that error was generally lower at depth positions closer to the fixation plane. For a plot of fit error versus *z* position showing all significant pairwise comparisons, see Extended Data Figure 6-2.

We next examined the amplitude of fits in each ROI, as an overall measure of representation robustness, or signal-to-noise ratio (Fig. 6*B*). In the *x* dimension, representation amplitude was similar in all areas, with the highest mean amplitude in V1 (mean [95% CI], 0.62 [0.61, 0.63]), and lowest amplitude in IPS0 (0.57 [0.56, 0.59]). In contrast, *z* representation amplitude was more variable across areas, with the highest amplitude in V3A (0.72 [0.63, 0.83]), followed by LO2 (0.69 [0.61, 0.80]), and the lowest amplitude in V3 (0.55 [0.50, 0.61]). We submitted the *z* representation amplitudes to a mixed effects analysis as previously described, which revealed a significant effect of ROI (χ^2^(8) = 40.89, *p* < 10^−5^), a significant effect of position (χ^2^(5) = 29.46, *p* < 10^−4^), but no interaction (χ^2^(40) = 41.43, *p* = 0.41). Pairwise comparisons revealed that amplitude was significantly higher in dorsal area V3A than in early visual areas V1, V2, V3, and V4 (Tukey corrected *p* < 0.05: V3A-V1 *t* = 0.14, SE = 0.04, *p* = 0.0040; V3A-V2 *t* = 0.17, SE = 0.04, *p* = 0.0002; V3A-V3 *t* = 0.17, SE = 0.04, *p* = 0.0001; V3A-V4 *t* = 0.14, SE = 0.04, *p* = 0.0073), and that amplitude was significantly higher in LO2 than in V2 and V3 (Tukey corrected *p* < 0.05: LO2-V2 *t* = 0.14, SE = 0.04, *p* = 0.0048; LO2-V3 *t* = 0.14, SE = 0.04, *p* = 0.0036). Across all ROIs, pairwise comparisons showed that amplitude was lowest at the furthest position (38.6 arcmin or –1.5 OpenGL units). For a plot of amplitude by *z* position showing all significant pairwise comparisons, see Extended Data Figure 6-2.

Finally, to provide a complementary measure of how well the model-based representations tracked the stimulus positions, we fit a line to the fit centers as plotted in Figure 5. This analysis is distinct from the SVM slope analysis shown in Figure 3 because the IEM allows us to test the accuracy of *x* or *z* encoding in various ROIs. A slope of 1 indicates perfect accuracy, while a slope of 0 would indicate that the spatial encoding model captured no information about stimulus depth at all. We found a significantly positive slope in the *x* centers of the model-based representations in every ROI examined, with many close to a slope of 1 (Fig. 6*C*, left panel). In the *z* centers of the model-based representations, we also found a significantly positive slope in all regions (Fig. 6*C*, right panel). While the *z* slope was highest in V3A and IPS0, statistical comparisons with other regions did not survive FDR correction (*p* < 0.05 uncorrected: IPS0-V1 0.29 [0.06, 0.53], *p* = 0.014; IPS0-V2 0.19 [0.02, 0.39], *p* = 0.022; V3A-V3 0.23 [0.02, 0.45], *p* = 0.028; IPS0-V3 0.26 [0.08, 0.43], *p* = 0.010).

## Discussion

The goal of this study was to examine how retinotopic regions of visual cortex encode the position-in-depth of a viewed stimulus. We presented subjects with stereoscopic, spherical stimuli at evenly gridded locations along both a horizontal (*x*) axis and a depth (*z*) axis that subtended a larger range of binocular disparities than sampled in most previous studies. We then used both decoding and encoding analysis approaches to assess how depth position is represented, using the horizontal positions as a reference point. Our decoding analyses revealed above chance decoding of *z* position in all retinotopic regions we examined, including V1–4, V3A, V3B, IPS0, and LO1–2. However, *z* decoding performance was highest in V3A, V3B, and IPS0, in agreement with the results of past studies (Goncalves et al., 2015; Finlayson et al., 2017). We also confirmed and extended past findings that decoding performance increased as two stimuli grew farther apart in disparity (Fig. 3; Preston et al., 2008). Most importantly, using an inverted encoding model, we were able to generate model-based representations of stimulus position along both the horizontal and depth axes. In both dimensions, the centers of the representations tracked the centers of viewed stimuli with reasonable accuracy, although *z* position representations were both less accurate and more variable than *x* position representations (Figs. 4–6). Comparing the amplitude of representations between ROIs provided evidence that the robustness of depth representations is higher in V3A than in early visual areas V1–V4. Taken together, these results support a model of spatial processing in which information about stimulus depth position emerges most prominently at an intermediate stage of the visual hierarchy (Backus et al., 2001; Tsao et al., 2003; Neri, 2004; Welchman et al., 2005; Preston et al., 2008; Durand et al., 2009; Georgieva et al., 2009; Minini et al., 2010; Goncalves et al., 2015; Welchman, 2016; Finlayson et al., 2017; Li et al., 2017).

Although our SVM and IEM analyses both suggested a key role for V3A in processing depth, they yielded slightly different conclusions about the strength of depth representations in other regions. Our SVM analysis (Fig. 2) indicated that V3B and IPS0 showed significantly higher discriminability of different depth positions than early visual areas, while the IEM analysis (Fig. 6) showed that model-based representation fit amplitudes were not significantly different between V1-V4 and either V3B or IPS0, although they were significantly different between LO2 and early visual areas V2 and V3. At the same time, although there were no significant differences in representation error across ROIs, IPS0 showed one of the lowest values of error, numerically more similar to V3A than to V1–V4. One interpretation of these results is that IEM error and decoding performance both measure the discriminability of depth representations, while representation amplitude measures the robustness, or signal-to-noise ratio, of depth representations. Based on this interpretation, the results may suggest that V3B and IPS0 represent depth with a high discriminability between different positions, while LO2 may have less discriminable but more robust representations. In any case, these analyses all suggest that representations in V3A are both more discriminable and more robust than representations in early visual cortex.

Although V3A was shown to represent depth the most strongly, both our decoding and encoding-model results show that the depth position of a stimulus is represented within a range of early (V1–V3), intermediate ventral (V4, LO1/2), and intermediate dorsal regions (V3A/B, IPS0) of visual cortex. This finding of widespread depth selectivity is in line with previous evidence from primate electrophysiology (Poggio et al., 1988; Hinkle and Connor, 2001; Tanabe, 2004; Trotter et al., 2004; Anzai et al., 2011; Hubel et al., 2015; Van Dromme et al., 2015), and univariate human fMRI analyses (Backus et al., 2001; Neri, 2004; Welchman et al., 2005; Bridge and Parker, 2007; Durand et al., 2009; Georgieva et al., 2009; Ip et al., 2014; Goncalves et al., 2015; Li et al., 2017). Further supporting the role of early visual areas in processing depth, human fMRI work shows that retinotopic visual responses in V1 are modulated by the perceived depth of objects (Murray et al., 2006). However, we also note that past work suggests disparity signals in the earliest regions of cortex (V1, V2) do not exhibit selectivity for correlated over anticorrelated disparity, which is a common test for whether disparity selectivity corresponds to perceived depth (Backus et al., 2001; Bridge and Parker, 2007; Preston et al., 2008; Ip et al., 2014; Goncalves et al., 2015). Therefore, we do not make a strong claim that all the regions we analyzed are directly associated with the perception of depth, and we interpret our results as confirming previous findings that selectivity for absolute binocular disparity is widespread in both dorsal and ventral visual areas.

In our study, we chose to evenly space the stimuli in rendered physical space to enable a direct comparison between the *x* and *z* IEM representations using identically structured encoding models and identically spaced stimuli. However, this necessarily meant that the stimuli were nonlinearly spaced by binocular disparity (Fig. 1). We speculate that mapping the *z*-axis by evenly spacing the stimuli by binocular disparity might yield more accurate representations of stimuli in depth. This could more effectively exploit any underlying structure in binocular disparity selectivity across cortex (Goncalves et al., 2015), although this remains a question for further empirical research.

An additional limitation of our design is that while we varied stimulus *x* position in addition to *z* position, we only had enough data to perform the SVM and IEM analyses separately for each axis. This means that the depth decoding and encoding analyses would rely heavily on voxels whose depth selectivity profile was tolerant to changes in horizontal position. This type of tolerance has been shown to increase along the posterior-anterior axis of the brain (Finlayson et al., 2017), so this could have resulted in an appearance of better depth representations in intermediate regions than early regions. Building a joint encoding model for *x* and *z* position, akin to a 2D encoding model for *x* and *y* position (Sprague and Serences, 2013; Ekman et al., 2017; Vo et al., 2017) would be one way for future studies to account for the interaction between *x* and *z* position encoding.

Finally, another limitation of the current study is that our stimulus presentation system did not allow for eye tracking in the scanner. Although subjects performed a demanding task at fixation, we cannot rule out the possibility of small changes in the vergence of the eyes toward the depth plane of the stimulus. One effect of these vergence movements would be to decrease the discriminability between depth positions, shifting representations of the furthest and nearest positions closer to the fixation plane. This may be one reason why our estimates of the slope between representation center and stimulus center in the *z* dimension are shallower than in the *x* dimension (Figs. 5, 6). This is also consistent with our finding that representation error in the *z* dimension was lowest at positions closest to the fixation plane (Extended Data Fig. 6-2).

Overall, these results provide evidence for tuned representations of disparity in multiple regions of retinotopic visual cortex, with strongest encoding in dorsal area V3A. Our decoding analysis demonstrated that the discriminability of stimuli scaled positively with disparity difference; this relationship was present widely throughout retinotopic cortex. In addition to confirming the findings of past work (Preston et al., 2008), we demonstrated that this scaling of decoding performance with disparity was present for extreme values of disparity up to +38 and –44 arcmin, as well as more moderate disparity values. Furthermore, our encoding model analysis allowed us to explicitly model a continuous depth axis. By constructing identical encoding models for both *x* and *z* position, we were able to compare the accuracy and robustness of depth position and horizontal position encoding. Although depth position was represented with overall lower accuracy and higher variability than horizontal position, we demonstrated that this technique can be used to recover representations of positions in depth. Our method is further validated by the fact that it recovered a similar pattern of performance across ROIs as a well-established decoding method. In future work, this technique may be used for other purposes such as characterizing the effects of spatial attention on 3D stimulus representations.

## Synthesis

Reviewing Editor: Siegrid Löwel, University of Goettingen Decisions are customarily a result of the Reviewing Editor and the peer reviewers coming together and discussing their recommendations until a consensus is reached. When revisions are invited, a fact-based synthesis statement explaining their decision and outlining what is needed to prepare a revision will be listed below. The following reviewer(s) agreed to reveal their identity: Julie Golomb.

The authors have done a good job revising their manuscript. While the main findings of the study are not particularly novel, as has been noted before, the paper will make a good contribution to the literature, confirming dorsal visual and parietal areas (V3A/V3B/IPS areas) as important processing modules for depth encoding.

Still, both reviewers' agree that minor revisions should be performed to clarify the few remaining issues, listed below:

1. Please clarify some of the new details about trial timing:

- It's stated that the stimulus is presented at the beginning of the trial for 3s, and then there's a 2-6s ITI. But the TR is 2s. Were the trial start times not synced with the TRs, or is there a missing 1s somewhere?

- To confirm, the single-trial activation estimates were not based on any GLM or deconvolution given the fast event-related design?

2. The stats on p.29-30 with all the pairwise comparisons are a bit hard to follow. It wasn't completely clear which data were being compared here any why - we are not sure all of these pairwise comparisons are necessary or are the best way to test for finer position effects, but if you do keep them, we would suggest referencing the corresponding figure earlier in the paragraph to help orient readers. Also, the pairwise stats here (and elsewhere in results) are given for conditions defined in arcmin, but the corresponding figures show the conditions defined in z-position. (This is extra challenging because far depths are negative z values but positive arcmin values.) Finally, we believe that starting at the bottom of p.29 it's for Z-position only, but this isn't stated.

3. As a final comment: One reviewer brought up the concern in the first round that vergence changes could contribute to decoding performance. The authors have some convincing arguments for why they believe this not to be the case. However, they argue that one consequence of vergence changes would be to reduce the effective disparity of the stimulus, making it more similar to a stimulus at the fixation plane. But - that is actually consistent with the pattern seen in many regions, e.g. in Fig 5, where the more extreme depths produce IEM fit centers closer to fixation than the true location. So we think it is “possible” there could be some small role of vergence here, and this could perhaps be acknowledged as a potential reason the fit slopes are shallower than actual.

## Author Response

We thank the reviewers for taking the time to provide detailed feedback on our manuscript. Below, we provide a point-by-point description of how we have modified the text to address each concern.

1. Please clarify some of the new details about trial timing: - It's stated that the stimulus is presented at the beginning of the trial for 3s, and then there's a 2-6s ITI. But the TR is 2s. Were the trial start times not synced with the TRs, or is there a missing 1s somewhere? - To confirm, the single-trial activation estimates were not based on any GLM or deconvolution given the fast event-related design?

Trial start times were not synced with TRs. We have added a sentence (pp.9) that explicitly states this. Single-trial activation estimates were not based on a GLM, and were obtained simply by taking an average of the signal at two timepoints (3rd and 4th TRs following stimulus onset). This method gives similar activation estimates to a GLM.

2. The stats on p.29-30 with all the pairwise comparisons are a bit hard to follow. It wasn't completely clear which data were being compared here any why - we are not sure all of these pairwise comparisons are necessary or are the best way to test for finer position effects, but if you do keep them, we would suggest referencing the corresponding figure earlier in the paragraph to help orient readers. Also, the pairwise stats here (and elsewhere in results) are given for conditions defined in arcmin, but the corresponding figures show the conditions defined in z-position. (This is extra challenging because far depths are negative z values but positive arcmin values.) Finally, we believe that starting at the bottom of p.29 it's for Z-position only, but this isn't stated.

We thank the reviewer for pointing out this confusing text. To avoid the need to switch between arcmin and OpenGL units, we have removed the sentences that list which pairwise comparisons were significant, and have instead put in a more general description stating that depth positions closer to fixation had a lower error (pp.29-30). We have made similar changes to the section where we describe representation amplitude (pp.31). All significant pairwise comparisons are still shown in graphical form in Extended Data Figure 6-2.

3. As a final comment: One reviewer brought up the concern in the first round that vergence changes could contribute to decoding performance. The authors have some convincing arguments for why they believe this not to be the case. However, they argue that one consequence of vergence changes would be to reduce the effective disparity of the stimulus, making it more similar to a stimulus at the fixation plane. But - that is actually consistent with the pattern seen in many regions, e.g. in Fig 5, where the more extreme depths produce IEM fit centers closer to fixation than the true location. So we think it is “possible” there could be some small role of vergence here, and this could perhaps be acknowledged as a potential reason the fit slopes are shallower than actual.

We agree with this point that vergence changes could be one reason why representation centers tend to be shifted toward the center of the space, resulting in shallower slopes. We have added a paragraph to the discussion (pp.37-38) acknowledging this.
