# Supplementary material for: Multivariate Analysis of BOLD Activation Patterns Recovers Graded Depth Representations in Human Visual and Parietal Cortex
Source: eNeuro. 2019 Jul 18;6(4):ENEURO.0362-18.2019. doi: 10.1523/ENEURO.0362-18.2019 (PMC6709213; doi:10.1523/ENEURO.0362-18.2019)
Supplement: Extended Data Table 1-1 — Actual values of position in OpenGL space, degrees visual angle, and disparity for each position in the stimulus grid Download Table 1-1, DOC file. [file sup_enu-eN-NWR-0362-18-s02.doc]

| Grid 1 (even runs) | | | |  | Grid 2 (odd runs) | | | |
| --- | --- | --- | --- | --- | --- | --- | --- | --- |
| Z position (OpenGL) | Disparity (arcmin) | X position (OpenGL) | X position (Degrees) |  | Z position (OpenGL) | Disparity (arcmin) | X position (OpenGL) | X position (Degrees) |
| -1.5 | 40.53 | -1.63 | -8.00 |  | -1.5 | 42.79 | -2.00 | -9.78 |
| -1.5 | 37.30 | -0.91 | -4.45 |  | -1.5 | 38.69 | -1.27 | -6.22 |
| -1.5 | 35.88 | -0.18 | -0.89 |  | -1.5 | 36.35 | -0.54 | -2.67 |
| -1.5 | 36.35 | 0.54 | 2.67 |  | -1.5 | 35.88 | 0.18 | 0.89 |
| -1.5 | 38.69 | 1.27 | 6.22 |  | -1.5 | 37.30 | 0.91 | 4.45 |
| -1.5 | 42.79 | 2.00 | 9.78 |  | -1.5 | 40.53 | 1.63 | 8.00 |
| -0.9 | 30.07 | -1.90 | -9.81 |  | -0.9 | 27.67 | -1.55 | -8.02 |
| -0.9 | 25.72 | -1.21 | -6.24 |  | -0.9 | 24.24 | -0.86 | -4.46 |
| -0.9 | 23.24 | -0.52 | -2.67 |  | -0.9 | 22.74 | -0.17 | -0.89 |
| -0.9 | 22.74 | 0.17 | 0.89 |  | -0.9 | 23.24 | 0.52 | 2.67 |
| -0.9 | 24.24 | 0.86 | 4.46 |  | -0.9 | 25.72 | 1.21 | 6.24 |
| -0.9 | 27.67 | 1.55 | 8.02 |  | -0.9 | 30.07 | 1.90 | 9.81 |
| -0.3 | 13.31 | -1.47 | -8.05 |  | -0.3 | 15.86 | -1.80 | -9.84 |
| -0.3 | 9.66 | -0.82 | -4.47 |  | -0.3 | 11.24 | -1.14 | -6.26 |
| -0.3 | 8.06 | -0.16 | -0.89 |  | -0.3 | 8.60 | -0.49 | -2.68 |
| -0.3 | 8.60 | 0.49 | 2.68 |  | -0.3 | 8.06 | 0.16 | 0.89 |
| -0.3 | 11.24 | 1.14 | 6.26 |  | -0.3 | 9.66 | 0.82 | 4.47 |
| -0.3 | 15.86 | 1.80 | 9.84 |  | -0.3 | 13.31 | 1.47 | 8.05 |
| 0.3 | -0.09 | -1.70 | -9.87 |  | 0.3 | -2.81 | -1.39 | -8.08 |
| 0.3 | -5.03 | -1.08 | -6.28 |  | 0.3 | -6.71 | -0.77 | -4.49 |
| 0.3 | -7.85 | -0.46 | -2.69 |  | 0.3 | -8.42 | -0.15 | -0.90 |
| 0.3 | -8.42 | 0.15 | 0.90 |  | 0.3 | -7.85 | 0.46 | 2.69 |
| 0.3 | -6.71 | 0.77 | 4.49 |  | 0.3 | -5.03 | 1.08 | 6.28 |
| 0.3 | -2.81 | 1.39 | 8.08 |  | 0.3 | -0.09 | 1.70 | 9.87 |
| 0.9 | -21.05 | -1.31 | -8.11 |  | 0.9 | -18.13 | -1.60 | -9.91 |
| 0.9 | -25.24 | -0.73 | -4.50 |  | 0.9 | -23.43 | -1.02 | -6.30 |
| 0.9 | -27.08 | -0.15 | -0.90 |  | 0.9 | -26.46 | -0.44 | -2.70 |
| 0.9 | -26.46 | 0.44 | 2.70 |  | 0.9 | -27.08 | 0.15 | 0.90 |
| 0.9 | -23.43 | 1.02 | 6.30 |  | 0.9 | -25.24 | 0.73 | 4.50 |
| 0.9 | -18.13 | 1.60 | 9.91 |  | 0.9 | -21.05 | 1.31 | 8.11 |
| 1.5 | -38.71 | -1.50 | -9.95 |  | 1.5 | -41.86 | -1.23 | -8.14 |
| 1.5 | -44.43 | -0.95 | -6.33 |  | 1.5 | -46.38 | -0.68 | -4.52 |
| 1.5 | -47.70 | -0.41 | -2.71 |  | 1.5 | -48.36 | -0.14 | -0.90 |
| 1.5 | -48.36 | 0.14 | 0.90 |  | 1.5 | -47.70 | 0.41 | 2.71 |
| 1.5 | -46.38 | 0.68 | 4.52 |  | 1.5 | -44.43 | 0.95 | 6.33 |
| 1.5 | -41.86 | 1.23 | 8.14 |  | 1.5 | -38.71 | 1.50 | 9.95 |
